# Species generalization and differences in Hedgehog pathway regulation of fungiform and circumvallate papilla taste function and somatosensation demonstrated with sonidegib

**DOI:** 10.1038/s41598-018-34399-3

**Published:** 2018-11-01

**Authors:** A. Kumari, Y. Yokota, L. Li, R. M. Bradley, C. M. Mistretta

**Affiliations:** 1 Department of Biologic and Materials Sciences, School of Dentistry University of Michigan Ann Arbor MI, 48109 Michigan, USA; 2 Department of Molecular and Integrative Physiology, Medical School University of Michigan Ann Arbor, MI 48109 Michigan, USA

## Abstract

Species generalization in the profound, modality-specific effects of Hedgehog pathway inhibition (HPI) in taste organ homeostasis and sensation is shown. With the HPI, cancer drug sonidegib, we demonstrate that the rat taste system, in addition to mouse, is regulated by Hedgehog signaling. After sonidegib treatment for 16–36 days in rat, there is loss of taste buds (TB) in soft palate, in fungiform (FP) and circumvallate papillae (CV), and elimination of taste responses from chorda tympani and glossopharyngeal nerves. The retained innervation in FP and CV during HPI cannot sustain TB. Responses to tactile stimuli are not altered, and temperature responses are reduced only after 28 days treatment, demonstrating modality-specific effects. Rat FP and neural effects are similar to those in mouse whereas TB and neural response effects from the rat CV are much more severe. When recovery is introduced in mouse after prolonged, 48 days HPI, the TB in CV are restored whereas those in FP are not. Overall, Hedgehog signaling regulation is shown to generalize to the rat taste system, and the modality-specific controls in taste organ sensation are affirmed. The reported, debilitating taste disturbances in patients who use HPI drugs can be better understood based on these data.

## Introduction

In mouse, we had demonstrated required roles for Hedgehog (HH) signaling in taste organ homeostasis and the elemental effects of HH pathway inhibition (HPI) on adult taste system integrity^1–3^. Taste buds (TB) in the fungiform papillae (FP) and taste responses from the chorda tympani (CT) nerve are eliminated after administration of the HPI drug sonidegib for 16 days in mouse^2,3^. Importantly, the functional effects are modality specific, because CT responses to tactile and temperature stimulation are retained.

However, although the HH pathway is a principal regulator of several organ systems^4^, the crucial effects of HH signaling in taste homeostasis have been shown in mouse only. The biology of a key signaling pathway in regulating taste organ integrity should be demonstrable beyond a single species. In addition, functional sensory effects in the mouse have been shown only for anterior tongue FP/TB/CT, but not studied for the soft palate, or the circumvallate (CV) papilla/TB responses from the glossopharyngeal nerve. Therefore, to fill crucial knowledge gaps we determined HPI drug effects in taste organs in the rat, an essential animal model for human physiology and disease. Genome editing techniques are emerging for rat^5–7^ and notably the physiological effects of HH signaling have been studied in several rat systems, including in ischemia and cardiomyocyte electrophysiology^8^, spinal cord regeneration^9^, and lung development^10^.

Depending on location, TB vary in innervation, density and exposure to the oral environment^11^. A single TB resides in surface epithelium of the rodent FP, directly exposed to oral stimuli via the taste pore, and innervated by the CT branch of the VIIth cranial nerve. On the soft palate, innervation is from another branch of the VIIth cranial nerve, the greater superficial petrosal, distributed to TB in rows, not in specialized papillae, in a mucosal tissue^12^. On the posterior tongue multiple TB are localized in the epithelium lining the clefts of foliate and CV papillae^13^, bathed by the secretions of von Ebner’s salivary glands. The CV TB are innervated by the glossopharyngeal (GL) nerve branch of the IXth cranial nerve. Therefore, study of FP and CV papillae, soft palate, TB, and neural responses after HPI addresses regulation of different taste systems by HH signaling.

In mouse there are effects of HPI not only after administration of the drug sonidegib but also after signaling inhibition in *Gli2* transgenic and gene deletion mice^1^, and *Smo* deletion models^3^. The HH pathway signals when the ligand binds to the transmembrane receptor PTCH1 and overrides PTCH-inhibition of Smoothened (SMO). SMO then signals to GLI transcription factors for expression of target genes that include *Ptch1 and Gli1*^14^. The HPI drug sonidegib blocks the HH pathway at SMO and is used to treat patients with basal cell carcinoma originating from deregulation of HH signaling^15–17^. Patients complain of taste dysgeusia and ageusia^18–20^, presumably from disruptions in taste papillae, TB and sensation^2,3^.

To test for HH signaling regulation of taste systems in rat, we gavaged animals with the HPI drug sonidegib, recorded from the CT or GL nerve, and determined effects on TB and on FP and CV structure, and on TB in soft palate. We compared rat CV effects to those in mouse CV/TB and responses from the GL nerve. Further we explored ability of the mouse taste system to recover from effects of prolonged HPI after discontinuing sonidegib administration for several months. In rat, HPI taste effects were extremely deleterious in FP and CV papilla systems, and in soft palate, but cold and touch responses were intact. Furthermore, effects in mouse CV/GL taste system were less profound than those in rat.

## Results

### TB in FP and taste responses are eliminated by HPI with sonidegib in rat

**Figure 1:**
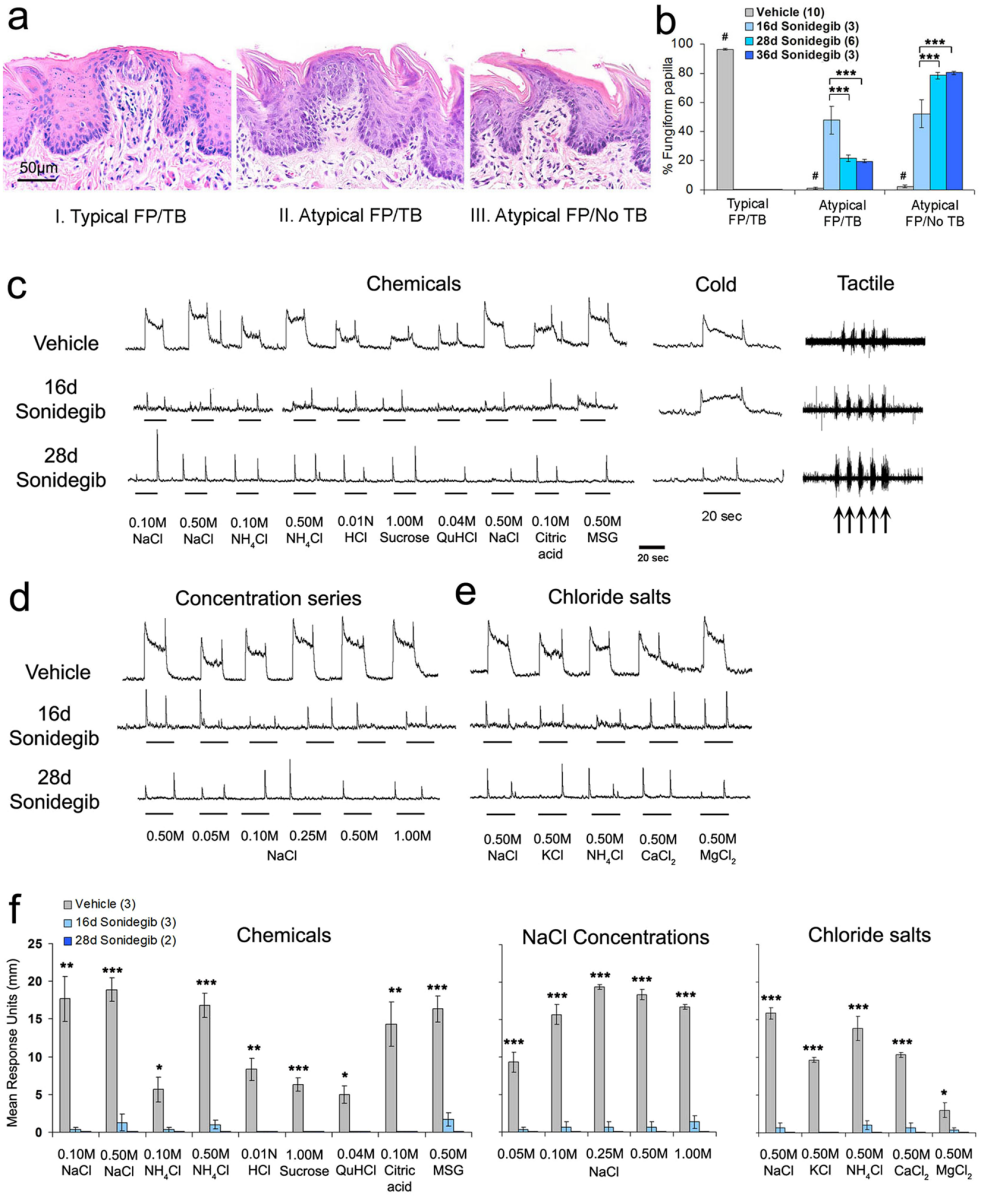
Hedgehog pathway inhibitor Sonidegib alters rat fungiform papilla (FP) and taste bud (TB) morphology, and reduces chorda tympani (CT) nerve responses to taste stimuli but not cold and tactile stimulation. (**a**) H&E staining illustrates FP and TB morphological categories: Typical FP/TB (I), Atypical FP/TB (II) and Atypical FP/No TB (III). Scale bar: 50μm, applies to all images. (**b**) Percentage of FP/TB categories indicates loss of Typical FP/TB. Bars represent mean ± SEM. Numbers in parentheses are the numbers of tongues analyzed per group. For each tongue 25–32 FP were analyzed. Vehicles for each duration are pooled for graphical representation only. Statistical analysis was two-way ANOVA_Duration X Treatment_ with Tukey HSD posthoc tests for duration. ^#^ denotes significant differences for Vehicle versus all Sonidegib treatments, at p < 0.001. Brackets indicate significant differences for treatment durations, ***p < 0.001. (**c**–**e**) CT nerve responses to lingual stimulation with Chemicals, Cold and Tactile stimuli (**c**), a Concentration series of NaCl (**d**) and high concentration Chloride salts (**e**), after Vehicle and 16d or 28d Sonidegib treatment. (**f**) Graph presents mean CT nerve responses (height of integrated response units in mm) to Chemicals, increasing NaCl concentrations, and Chloride salts. Bars represent mean ± SEM. Numbers in parentheses are numbers of rat analyzed per group for (**c**–**f**). Asterisks denote significant differences between Vehicle and both Sonidegib treatments (*p ≤ 0.05; **p ≤ 0.01; ***p ≤ 0.001).

The same animals were used for histology, electrophysiology and immunostaining. In histological analysis, begun after electrophysiology, we categorized FP and TB in three types: Typical FP/TB (Type I), Atypical FP with TB remnants (Type II), and Atypical FP with no TB (Type III) (Fig. 1a). The Type 3 Atypical FP/No TB, somewhat resembles the filiform papilla but can be differentiated by a wider, taller stromal core than the filiform and a papilla apex that is not usually spinous. We present effects on each FP/TB Type over treatment times (Fig. 1b). The HPI cancer drug sonidegib was gavaged for 16–36d, and there was a complete loss of Typical FP/TB (Type I) after only 16d [F(3,22) = 9712, p < 0.001)], with a gradual increase in Atypical FP with no TB (Type III) [F(3,22) = 173, p < 0.001)]. Some Atypical FP with TB remnants (Type II) were retained even after 36d treatment [F(3,22) = 34.3, p < 0.001)]. Whereas papilla types were altered, the overall density of FP was maintained across 16–36d treatment compared to Vehicle [range from 25 to 32 FP per 800 µm standardized tongue region; F(3,22) = 1.8, p = 0.19] and filiform papillae were intact. To test anterior tongue TB function after 16–28d HPI, CT nerve responses were recorded to a range of chemical stimuli. Responses were virtually eliminated after 16d and no responses were observed after 28d (Fig. 1c). Nor were there responses to a range of NaCl concentrations (Fig. 1d) or a series of chloride salts at high concentration (Fig. 1e). Notably, CT nerve responses to tactile stimulation were maintained for 28d of sonidegib treatment as were responses to cold water through 16d of HPI (Fig. 1c). By 28d sonidegib gavage, responses to cold were almost eliminated. The results demonstrate a rapid, severe effect of HPI in rat FP/TB and a modality specific effect in CT recordings. Quantified responses are in Fig. 1f: for chemical stimuli [F(2,27) = 8.1–72.1, p = 0.000–0.027], NaCl concentrations [F(2,27) = 34.5–504.5, p ≤ 0.001], and chloride salts [F(2,27) = 8.7–657.0, p = 0.000–0.024].

**Figure 2:**
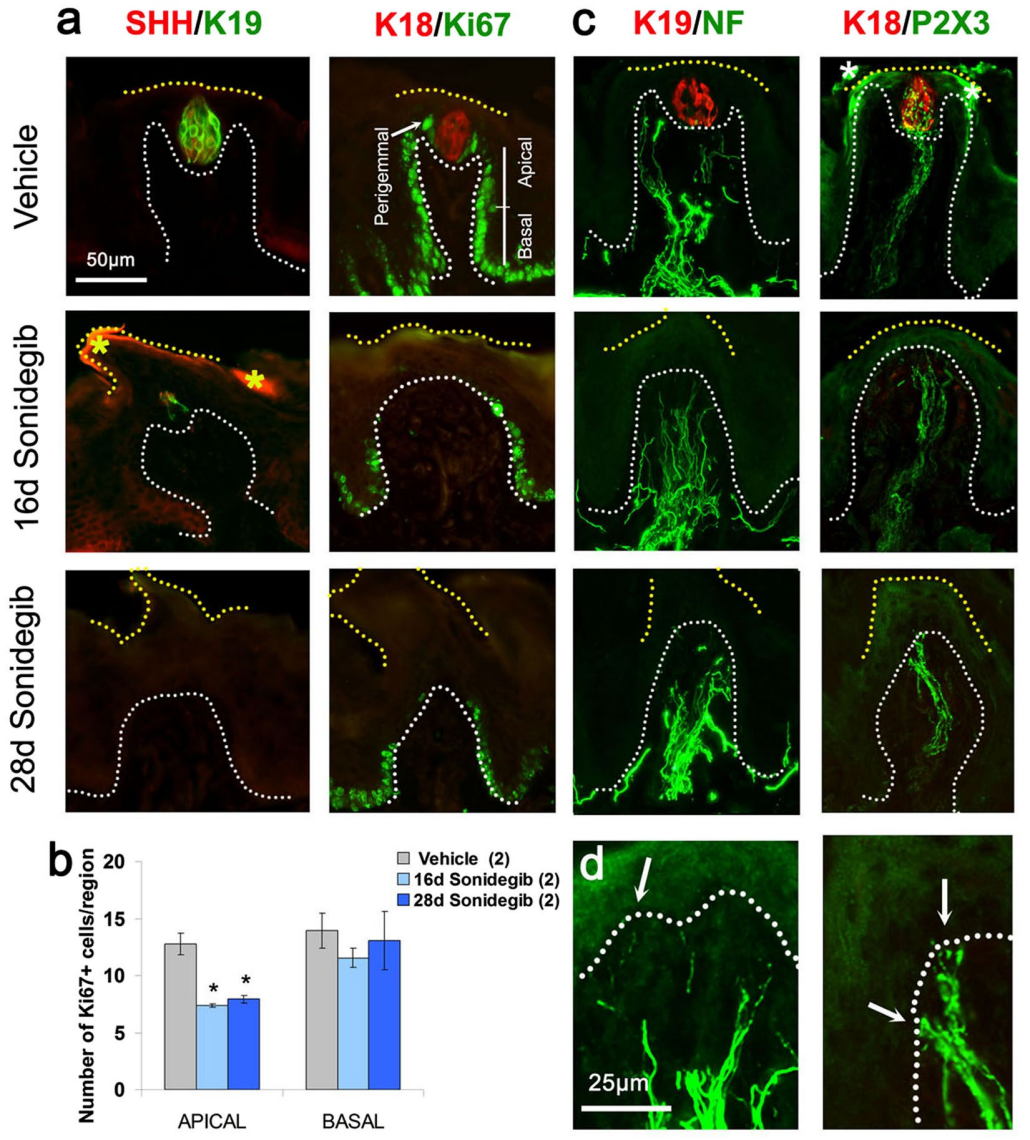
Sonidegib treatment reduces taste buds (TB), SHH ligand and proliferation in rat fungiform papilla (FP) while innervation is retained. (**a**) Immunofluorescent antibody detection of SHH ligand (red) and K19 (green) for TB cells; K18 (red) for TB cells and Ki67 (green) for cell proliferation, after Vehicle, 16d or 28d Sonidegib treatments. SHH is reduced in association with TB, K19+ cell loss. Asterisks (*) indicate nonspecific SHH immunoproduct in cornified surface cells in 16d Sonidegib image. The Vehicle, K18/Ki67 image shows 3 regions positive for Ki67+ cells (Apical, Basal and Perigemmal). Proliferating cells are lost in Apical FP region after 16–28d Sonidegib. (**b**) Number of Ki67+ cells in Apical and Basal regions of FP in Vehicle- and Sonidegib-treated mice. Numbers of tongues analyzed are in parentheses. For each tongue 8–10 FP were analyzed. Sonidegib treatment reduces apical epithelial cell proliferation in FP compared to Vehicle. Statistical analysis was one-way ANOVA with Tukey HSD posthoc comparisons (*p ≤ 0.05, compared to Vehicle, APICAL). (**c**) Immunofluorescent antibody detection of K19 or K18 (red) for TB cells and NF (green) for lingual and CT innervation or P2X3 (green) for CT nerve fibers. Innervation was retained after Sonidegib exposure. Asterisks (*) indicate nonspecific P2X3 immunoproduct in surface layer in Vehicle image. (**d**) Enlarged images from 28d Sonidegib papillae. Arrows point to NF+ or P2X3+ fibers in the FP epithelium. Throughout, white dotted lines indicate the basal lamina. Yellow dotted lines indicate surface of epithelium. (**a**,**c**) Scale bar: 50 μm, applies to all images. (**d**) Scale bar: 25 μm, applies to both images.

In FP epithelium SHH is restricted to TB cells. Therefore, with loss of TB there was an associated loss of epithelial SHH ligand (Fig. 2a, SHH/K19). We assessed cell proliferation and observed Ki67+ proliferating cells in the basal epithelial layer of the FP walls with Vehicle treatment (Fig. 2a, K18/Ki67). Because HH regulation of proliferation in mouse FP has been reported^3,21^ we counted Ki67+ cells in FP apical and basal wall regions (Fig. 2a, Vehicle). Proliferating cells in the epithelial compartment (Fig. 2b) were reduced in the apical half of FP walls [F(2,5) = 24.9,p = 0.01] but not in the basal half [F(2,5) = 0.45,p = 0.67]. There were too few TB remaining to quantify perigemmal cells.

Whereas TB cells are dependent on an intact innervation^22^, after loss of TB in rats that had been gavaged for up to 28d a robust innervation was retained in FP, including lingual nerve fibers (Fig. 2c, K19/NF) and P2X3-labeled fibers of the CT nerve (Fig. 2c, K18/P2X3). Nerves were in close association with the basal lamina region of the apical FP, at the former location of TB in the epithelium (Fig. 2c). However, as in mouse, even in the context of intact innervation, the TB cannot be maintained in an epithelium with disrupted HH signaling^1–3^.

### TB in soft palate eliminated by sonidegib treatment in rat

**Figure 3:**
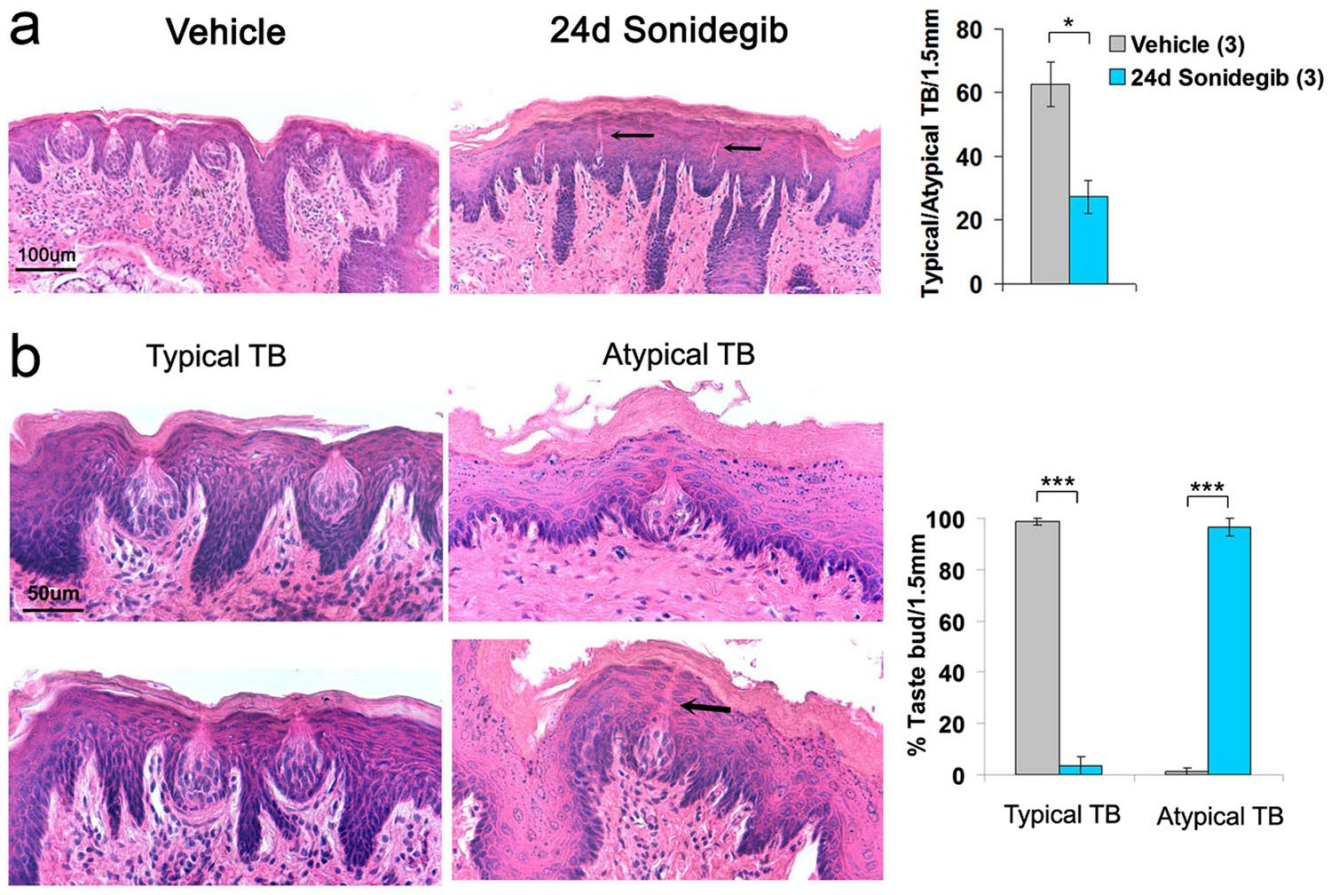
Sonidegib alters rat soft palate taste bud (TB) morphology and numbers. (**a**) H&E staining illustrates row of TB after Vehicle or 24d Sonidegib treatment. The number of TB seen after 24d Sonidegib is reduced. Arrows indicate TB remnants with long thin pores. Scale bar: 100 μm, applies to both images. The graph demonstrates that the number of Typical plus Atypical TB is reduced by about half in Sonidegib-treated rats. Bars represent mean ± SEM. Numbers in parentheses are the numbers of soft palates analyzed per group. (*p < 0.05; t(4) = 3.33, p = 0.03). (**b**) H&E staining illustrates Typical TB in Vehicle and Atypical TB in Sonidegib treatments. The arrow points to a remaining taste pore ‘path’ in an Atypical TB. Scale bar: 50 μm, applies to all images. Percentage of TB categories is presented in graph. Bars represent mean ± SEM. Typical TB are almost eliminated and Atypical TB are at almost 100% of total. ***p < 0.001, Sonidegib treatment compared to Vehicle; t-tests: t(4) > 24.00.

Soft palate TB reside within a stratified epithelial mucosa, not in specialized papilla (Fig. 3a, Vehicle). These TB have not previously been analyzed after HPI. We observed TB remnants with long thin taste pores after 24d sonidegib treatment (Fig. 3a, 24d Sonidegib, arrows point to ‘pores’ from TB remnants). Overall, after HPI there were reduced numbers of *total* TB (Typical TB with a pore plus Atypical TB remnants) in the Geschmacksstreifen region (Fig. 3a, Graph for TB per 1.5 mm). Furthermore, after HPI the epithelium was apparently thickened (Fig. 3b, Vehicle and 24d Sonidegib) and for some of the Atypical TB, traces were seen of former sites where the taste pores had been located (Fig. 3b, 24d Sonidegib, bottom, Atypical TB, arrow). The effect was substantial with Typical TB virtually eliminated after HPI and about 100% of TB were Atypical (Fig. 3b, graph). Thus, in the soft palate, HH signaling regulates TB maintenance, demonstrated with reduced overall TB or remnant count and typical TB elimination after HPI.

### TB in CV and taste responses are essentially eliminated by sonidegib treatment in rat

**Figure 4:**
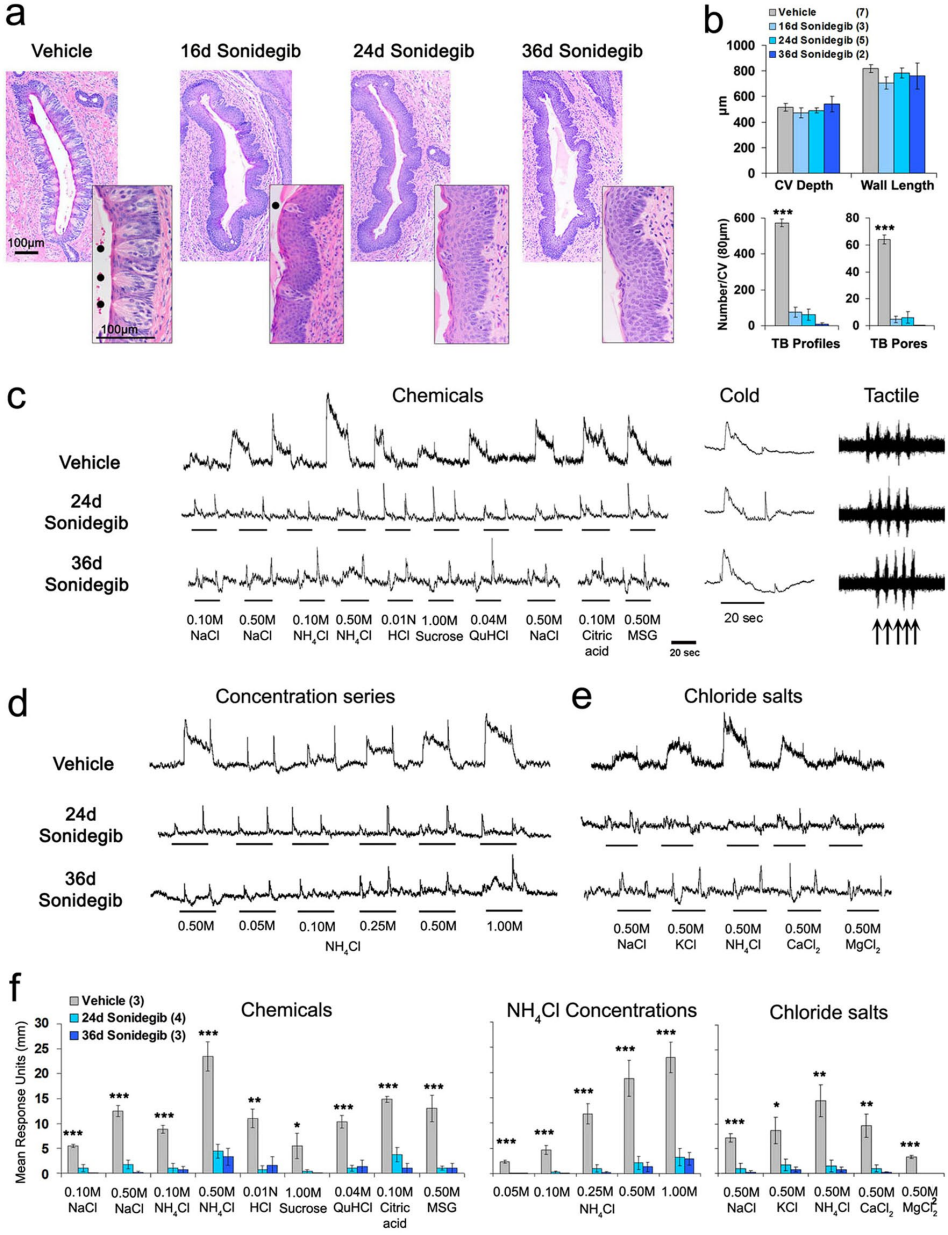
Sonidegib treatment in rat eliminates taste buds (TB) in the circumvallate papilla (CV) and glossopharyngeal (GL) nerve responses to lingual stimulation with taste stimuli but not cold or tactile stimuli. (**a**) H&E staining illustrates one wall of the CV and TB in rats treated with Vehicle or 16–36d with Sonidegib. Insets are enlarged images of CV epithelium to illustrate TB morphology. Dots point to TB Pore in Vehicle or after 16d Sonidegib. Scale bars: 100μm, apply to all images or insets. (**b**) Graphs for CV depth and wall length indicate no changes in papilla structure. However, TB Profiles and TB Pores were significantly and substantially decreased at all exposure periods relative to Vehicle. Bars represent mean ± SEM. Numbers in parentheses in the legends are numbers of tongues analyzed per group. Statistical analysis was one-way ANOVA with Tukey HSD posthoc comparisons. *** denote significant differences for Vehicle versus all Sonidegib treatments, at p < 0.001. (**c–e**) GL nerve responses to lingual stimulation with Chemicals, Cold and Tactile stimuli (**c**), a Concentration series of NH_4_Cl (**d**), and high concentration Chloride salts (**e**) after Vehicle and 24 or 36d Sonidegib treatment. Underlines denote when the respective stimulus was applied. We note that chemical responses at 24d and 36d Sonidegib treatment have a baseline that reflects stimulus and on- and off-set elements more prominently in the absence of robust taste responses. (**f**) Graph presents mean GL nerve responses (height of integrated response units in mm) to Chemicals, increasing NH_4_Cl Concentrations, and Chloride salts. Bars represent mean ± SEM. Numbers in parentheses are numbers of rat analyzed per group. Asterisks denote significant differences between Vehicle and both Sonidegib treatments (*p ≤ 0.05; **p ≤ 0.01; ***p ≤ 0.001).

The same animals were used for histology, electrophysiology and immunostaining. In histological analysis, begun after electrophysiology, we assessed CV structure and TB. Examining CV size (Fig. 4a,b), we did not find differences in papilla depth [F(3,16) = 0.49, p = 0.69] or wall length [F(3,16) = 1.2, p = 0.35] in rats treated with sonidegib up to 36d compared to vehicle. However TB pores (Fig. 4a,b) were almost eliminated [F(3,16) = 72.7, p < 0.001] as were responses from the GL nerve (Fig. 4c,d,e,f) to a range of chemical stimuli [F(2,9) = 5.0–58.9, p = 0.000–0.025], a concentration series of NH_4_Cl [F(2,9) = 19.8–60.0, p ≤ 0.001] or high concentration chloride salts [F(2,9) = 6.6–122.5, p = 0.000–0.025]. Very small responses did remain to 0.50 M salts and 0.50 and 1.00 M NH_4_Cl. Importantly, however, both tactile and temperature sensation was retained and robust after 36d drug exposure, demonstrating modality-specific effects after HPI.

**Figure 5:**
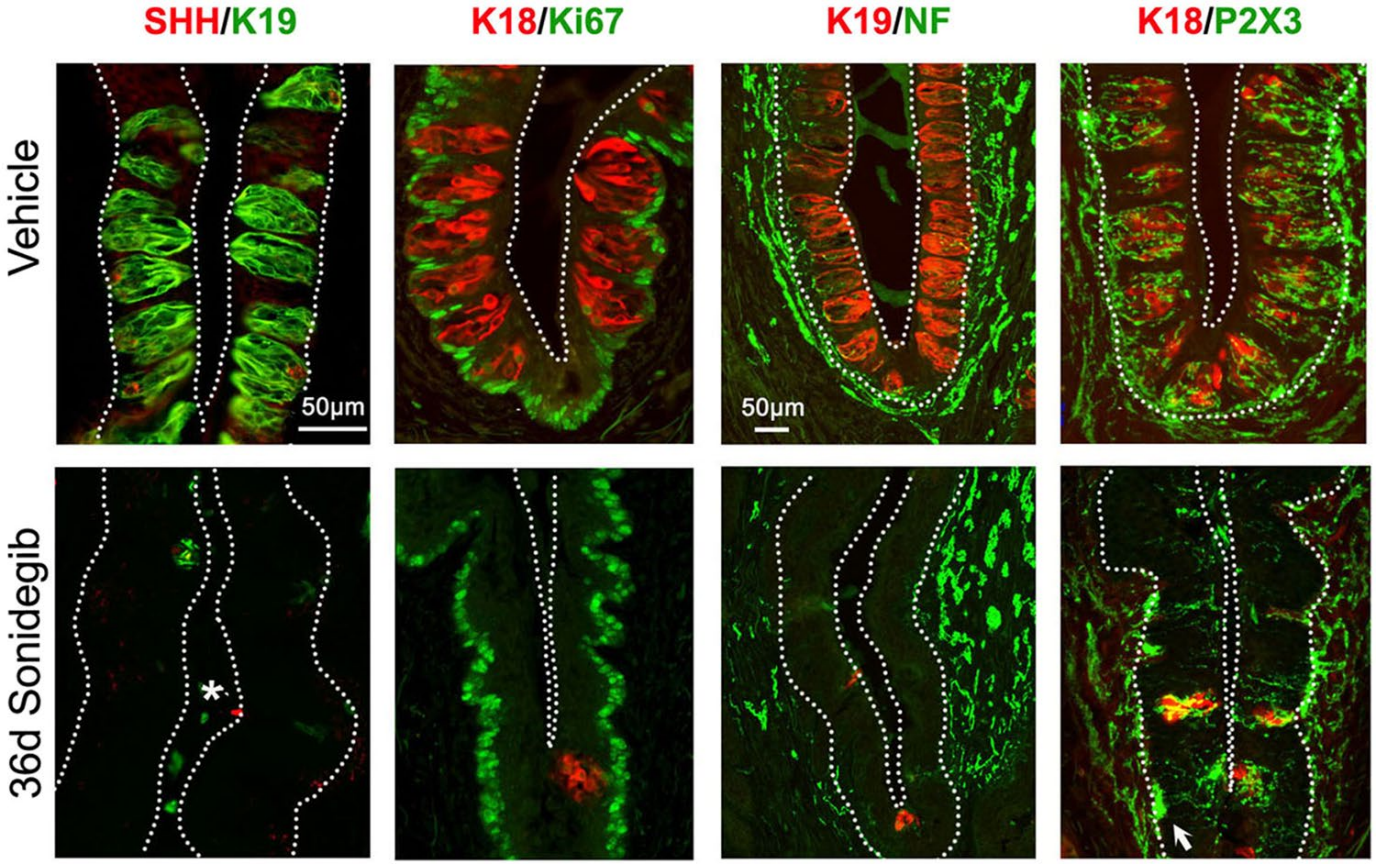
Sonidegib treatment in rat reduces taste buds (TB) and SHH ligand in circumvallate papilla (CV) whereas cell proliferation and GL innervation are retained. Immunofluorescent antibody detection of SHH ligand (red) and K19 (green) for TB cells; K18 (red) for TB cells and Ki67 (green) for cell proliferation; K19 (red) for TB cells and NF (green) for innervation; and, K18 (red) for TB cells and P2X3 (green) for taste nerve fibers, after Vehicle or 36d Sonidegib treatment. White dotted lines outline the epithelium. Asterisk in SHH/K19, 36d Sonidegib indicates nonspecific K19 immunostaining. Arrow points to the P2X3+ nerves extending into CV epithelium after Sonidegib treatment. SHH is reduced in association with TB cells. Cell proliferation is maintained and nerves fibers are retained after Sonidegib treatment. Scale bar: 50μm, applies to all images, except K19/NF.

As in the FP, SHH expression is reduced/eliminated in CV epithelium associated with TB elimination (Fig. 5, SHH/K19). On examining the epithelial basal cells throughout the CV there was no obvious loss in Ki67+ cell density (Fig. 5, K18/Ki67, Vehicle and 36d Sonidegib). Whereas only a few remnants of TB remained, the CV innervation identified with NF and P2X3 (taste specific) immunoreactions was retained after HPI for 36d (Fig. 5, K19/NF; K18/P2X3). We did not quantify the amount of innervation and there is of course some variability; however, robust innervation is obvious in the CV complex and was observed across sonidegib-treated tongues. Nerves that remained were close to basal lamina regions and could extend into the CV epithelium (Fig. 5, 36d Sonidegib, K18/P2X3 arrow).

### TB in CV and taste responses are reduced by sonidegib treatment in mouse

**Figure 6:**
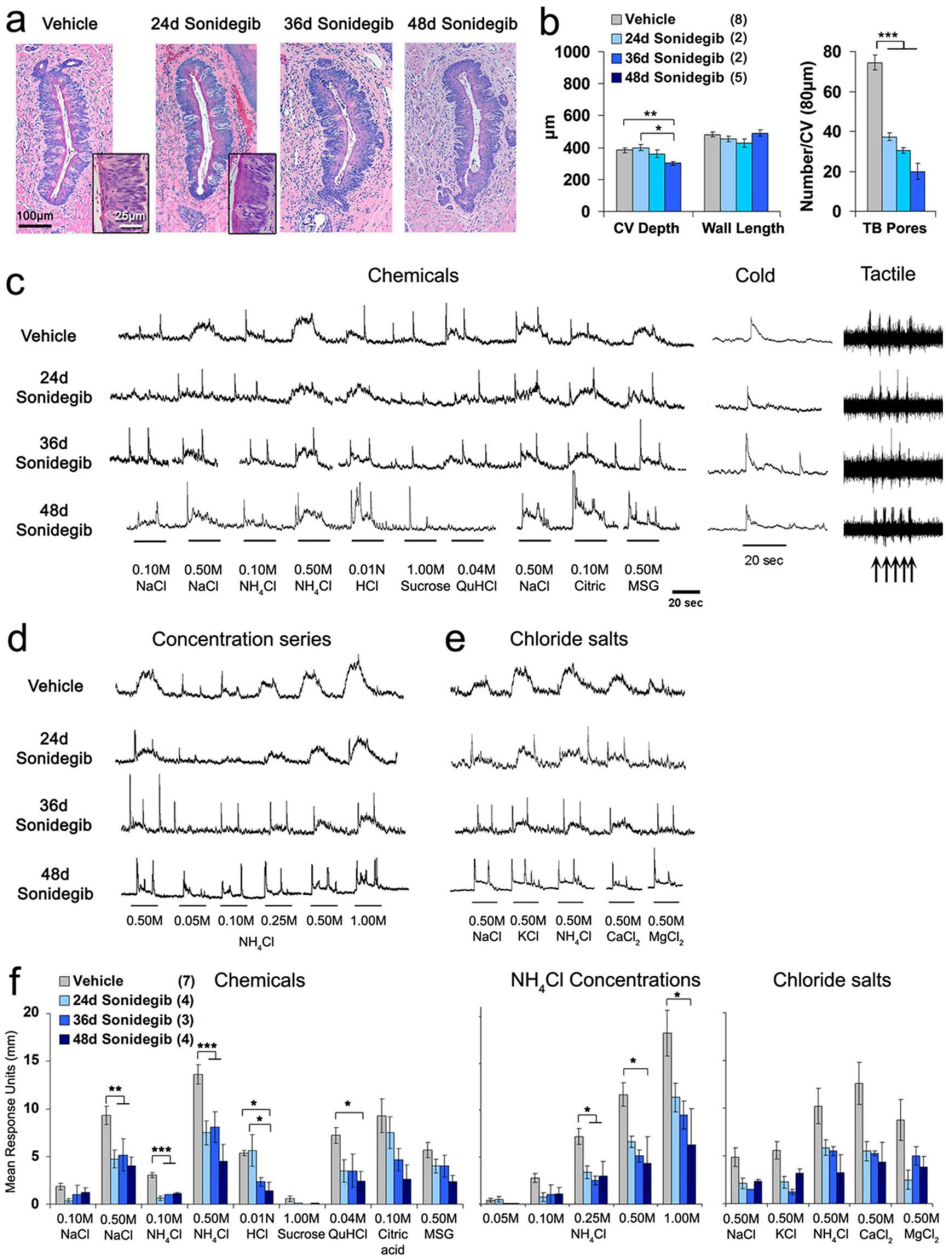
Sonidegib treatment in mouse reduces taste buds (TB) in the circumvallate papilla (CV) and glossopharyngeal (GL) nerve responses to lingual stimulation with chemicals, but not to cold or tactile stimuli. (**a**) H&E staining illustrates one wall of CV and TB in Vehicle and Sonidegib treated mice. Insets are enlarged images to illustrate TB morphology. TB are reduced with Sonidegib treatment. Scale bar: 100μm, applies to all images. Scale bar: 25 μm, applies to both insets. (**b**) Graphs for CV depth and wall length and number of TB pores in Vehicle and after Sonidegib treatment. Bars represent mean ± SEM per group. Numbers in parentheses in the legend are numbers of tongues analyzed per group. Statistical analysis was one-way ANOVA with Tukey HSD posthoc comparisons (*p ≤ 0.05; **p ≤ 0.01; ***p ≤ 0.001). Brackets indicate significance between treatments. CV depth was significantly but very modestly reduced, 48d Sonidegib compared to Vehicle (**p ≤ 0.01) and to 24d Sonidegib (*p ≤ 0.05). However, TB Pores/CV region were significantly decreased by more than half. ***Denote significant differences for Vehicle versus all Sonidegib treatments, at p ≤ 0.001. (**c–e**) GL nerve responses to lingual stimulation with Chemicals, Cold or Tactile stimuli (**c**), a Concentration series of NH_4_Cl (**d**), and high concentration Chloride salts (**e**), after Vehicle or 24–48d Sonidegib treatment. Substantial taste responses remained after 48d treatment. Underlines denote periods of stimulus application. (**f**) Graph presents mean GL nerve responses (height of integrated response units in mm) to Chemicals, increasing NH_4_Cl Concentrations, and Chloride salts. Brackets denote significant differences between treatments (One-way ANOVA with Tukey posthoc tests, *p ≤ 0.05; **p ≤ 0.01; ***p ≤ 0.001). Responses to salts and acid stimuli were reduced by only about 50–60%. Bars represent mean ± SEM. Numbers in parentheses are numbers of mice analyzed per group.

We had reported that TB numbers are reduced in the mouse CV after sonidegib treatment for 36d^3^. However, compared to the reduction in FP/TB in mouse after HPI, which was a complete elimination, the reported loss in CV/TB was modest, with about 40% of TB remaining, compared to vehicle-treated. To learn how remaining CV/TB functioned in taste responses, and to compare with rat, we recorded from the GL nerve in mice treated with Vehicle or 24, 36 and 48d sonidegib. Because the 48d treatment was a considerable extension to the prior 36d HPI with sonidegib, we first analyzed CV and TB to determine effects after prolonged treatment (Fig. 6a,b). Whereas papilla wall length was maintained [F(3,17) = 1.6, p = 0.24] and CV depth was very modestly decreased [F(3,17) = 6.4, p = 0.006], the number of TB pores was substantially reduced [F(3,17) = 43.4, p < 0.001]. However, even after 48d HPI, about 25% of Vehicle TB number remained, assessed by taste pore counts (Fig. 6b, TB Pores). This contrasts with study of the FP in mouse after HPI, with a complete loss of TB^2^ (and replicated in the current paper).

In the mouse GL nerve (Fig. 6c–f), after HPI for 24–48d there were modest to moderate responses to all chemicals, compared to Vehicle [F(3,17) = 1.8–23.4, p = 0.000–0.20], including to a concentration series of NH_4_Cl [F(3,15) = 1.1–5.6, p = 0.01–0.40], and to a series of high concentration chloride salts [F(3,15) = 2.2–4.5, p = 0.02–0.14]. Notably, responses to tactile and temperature stimuli were robust throughout HPI treatment durations (Fig. 6c). Therefore HPI effects with sonidegib treatment on CV/TB and GL nerve responses in mouse were substantial but relatively modest compared to those in rat. For example, in mouse the responses to 0.50M NaCl or NH_4_Cl were reduced by about 50% compared to Vehicle, but in rat they were effectively eradicated. Furthermore, even after prolonged sonidegib treatment, for 48d, TB remained in the mouse CV and taste responses still were recorded from the GL nerve.

**Figure 7:**
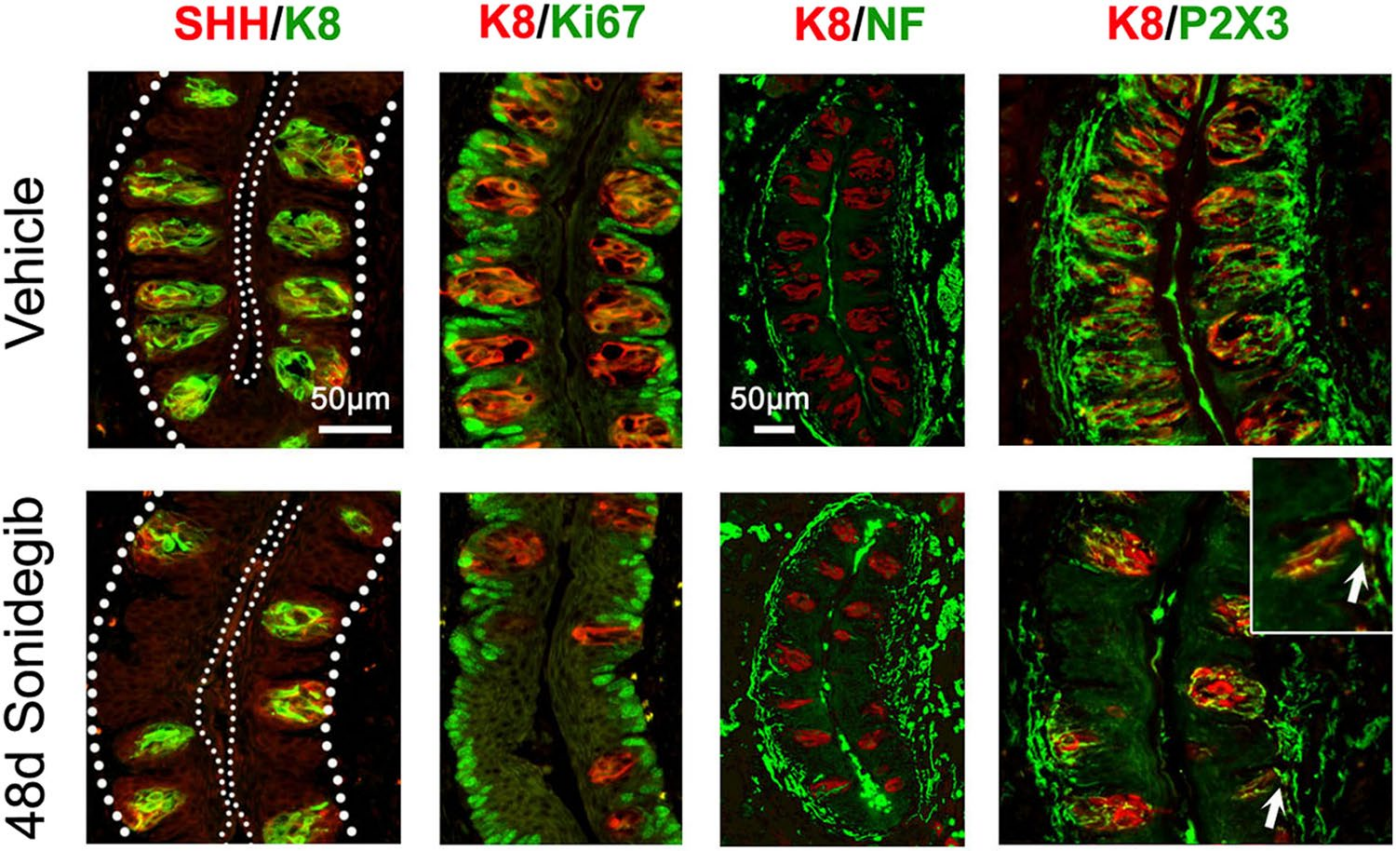
Long term Sonidegib treatment in mouse reduces taste buds (TB) and SHH ligand in circumvallate papilla (CV) whereas proliferation and innervation are retained. Immunofluorescent antibody detection of SHH ligand (red) and K8 (green) for TB cells; K8 (red) for TB cells and Ki67 (green) for cell proliferation; and K8 (red) with NF (green) for GL innervation or P2X3 (green) for GL taste fibers, after Vehicle or 48d Sonidegib treatment. For SHH/K8, large dotted lines indicate the basal lamina. Small dotted lines outline the surface epithelium. Inset (K8/P2X3) shows an image of nerves extending into CV epithelial basal lamina (arrow). Scale bar: 50 μm, applies to all images. Inset at 2×.

The SHH ligand, restricted to TB in the CV, was reduced in mouse in association with reduced numbers of TB after HPI (Fig. 7, SHH/K8 48d Sonidegib). In contrast to data reported for the CV after HH suppression in *Gli2* genetic deletion mice^1^, there was not an apparent reduction in Ki67+ proliferating cells in the basal layer of the mouse CV papilla epithelium after sonidegib treatment (Fig. 7, K8/Ki67). Further, robust innervation is obvious in the CV complex even after 48d HPI with sonidegib (Fig. 7, K8/NF; P2X3/K8). Nerves remaining after HPI were apposed to the CV basal lamina and some entered into the papilla epithelium (Fig. 7, P2X3/K8 inset).

### Recovery from prolonged HPI in mouse FP/TB and CV/TB

The exposure to sonidgeib for 48d in mice was a prolonged HPI treatment. We had reported that the FP/TB could recover partially from 16d sonidegib treatment^3^. That is, after discontinuing the drug for up to 9mo, about 50% of Vehicle-treated numbers had regenerated. On the other hand, recovery in the mouse CV/TB after 16d sonidegib treatment was complete, with a return to the same TB numbers as Vehicle-treated mice, at 3–9mo after discontinuing the drug^3^.

**Figure 8:**
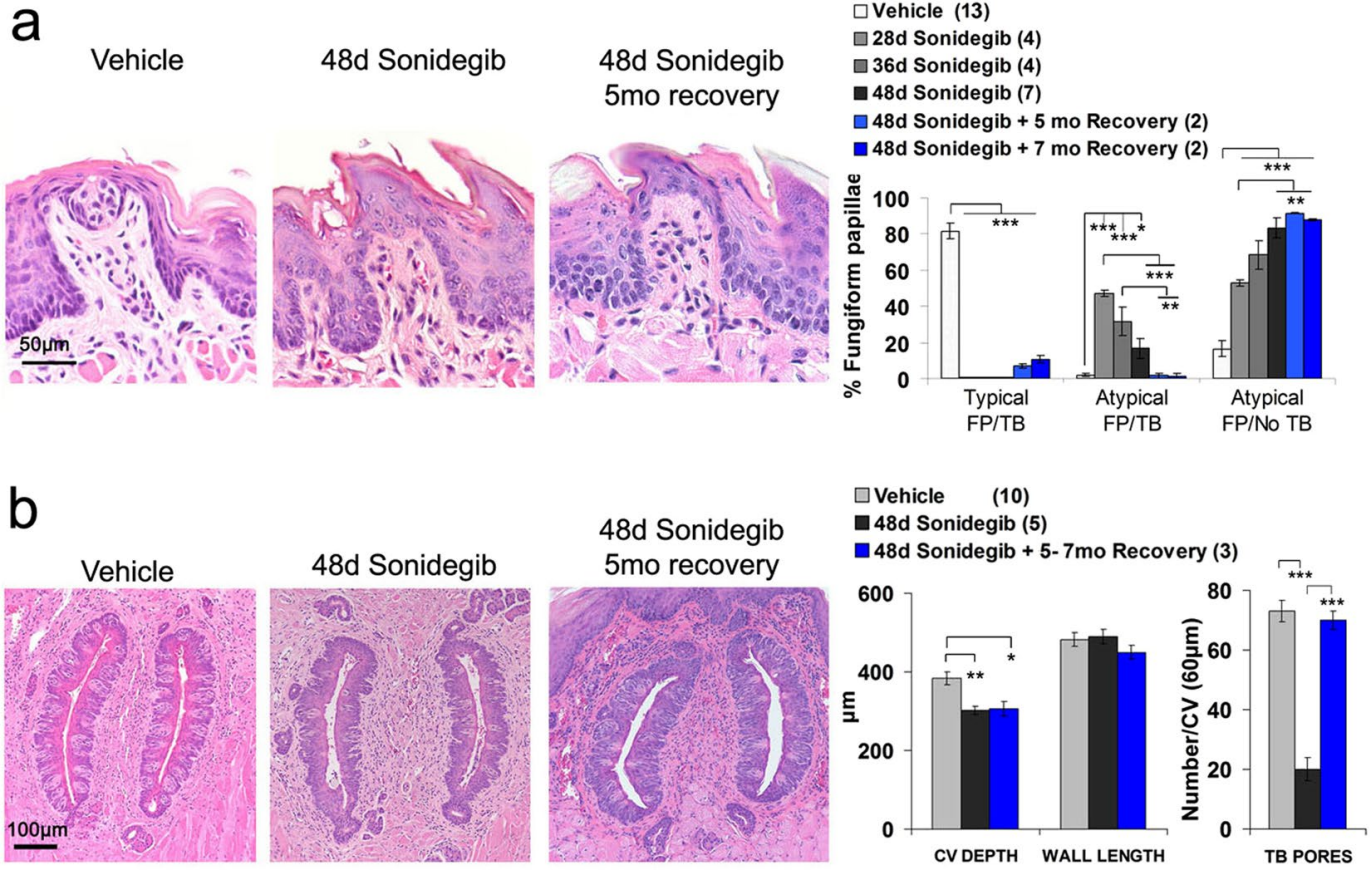
Cessation of extended Sonidegib treatment in mouse results in recovery of circumvallate taste bud pores (CV/TB) but not fungiform papilla taste bud (FP/TB) morphology. (**a**) H&E staining illustrates FP and TB morphology after Vehicle and 48d Sonidegib treatment, and 48d Sonidegib treatment with 5 months of recovery. Scale bar: 50 μm, refers to all images. Graph shows percentage of Typical FP/TB, Atypical FP/TB and Atypical FP/No TB after 28, 36 or 48 days of Sonidegib treatment, and 48d Sonidegib followed by Recovery periods of 5 and 7 months. Bars represent mean ± SEM. Numbers in parentheses are numbers of mouse tongues. For each tongue 32 to 43 FP were analyzed. Vehicles for each duration are pooled for analysis. Statistical analysis: One-way ANOVA with Tukey HSD posthoc tests. Typical FP/TB: F(5,31) = 264.5, p < 0.001; Atypical FP/TB: F(5,31) = 19.9, p < 0.001; Atypical FP/No TB: F(5,31) = 53.1, p ≤ 0.001. Brackets denotes significant differences between groups (*p ≤ 0.05; **p ≤ 0.01; ***p ≤ 0.001). (**b**) H&E staining to illustrate morphology of CV/TB after Vehicle and 48d Sonidegib treatment, and Recovery at 5 months after stopping treatment. Scale bar: 100 μm, refers to all images. Graphs illustrate CV structure measures and TB pores/CV region. Bars represent mean ± SEM. Numbers in parentheses are numbers of mouse tongues. Compared to Vehicle, the CV depth was reduced after 48 Sonidegib treatment (**p ≤ 0.01) and did not reach full recovery but was still reduced at 5–7 mo Recovery periods (*p ≤ 0.05). [Analysis: One-way ANOVA with Tukey HSD posthoc comparison: F(2,17) = 7.0, p = 0.007]. Wall length did not change after Sonidegib treatment or during recovery [F(2,17) = 0.71, p = 0.51]. TB pores were reduced in 48d Sonidegib-treated mice and restored completely within 5–7mo Recovery. Brackets indicate significant differences [***p ≤ 0.001; One-way ANOVA with Tukey HSD posthoc comparison, F(2,17) = 46.6, p < 0.001].

To learn if recovery still would occur after the 48d sonidegib treatment, we studied mice maintained for 5 or 7mo with no drug. In the FP/TB there was essentially no recovery of Typical FP/TB, with up to about 90% Atypical FP/No TB papillae persisting on the tongue (Fig. 8a). In contrast, in the CV there was recovery of TB with pores back to numbers of Vehicle treatment after 5–7mo with no drug, even though the HPI treatment had been prolonged (Fig. 8b, TB Pores). These data suggest differences in recovery potential between anterior and posterior taste organs. If HPI has eliminated all TB, as in FP/TB effects, there is virtually no recovery of TB, whereas if TB remain after HPI, as in the CV, then recovery takes place even after 48d sonidegib treatment.

## Discussion

Regulatory roles for HH signaling in taste homeostasis had been reported only in mouse, although proposed translational applications to human patients using HPI drugs are numerous^2,3,23^. To test for generalization of biological principles in taste regulation and to fully understand potential validity in application to human taste homeostasis, studies beyond mouse were necessary. We used the cancer treatment drug sonidegib^24^ to treat adult rats and tested hypotheses about roles for HH signaling in TB maintenance. We report that the rat taste system is regulated by HH signaling, and is extremely sensitive to HPI, formalizing the pathway as essential in taste organ homeostatic control across two species. We have summarized and compared sonidegib effects on oral taste buds in rat and mouse in Table 1. The rat is established as a robust model for studying HH signaling in taste organs.

**Table 1:** Taste system effects of HH Pathway Inhibition (HPI) with sonidegib.

Taste Effect	Rat	Mouse
**Fungiform papilla (FP) and Chorda Tympani (CT) Nerve Responses**		
FP/TB morphology	FP acquire conical keratinized apex, loss of TB	FP acquire conical keratinized apex, loss of TB^2,3^ (and Fig. 8 this paper)
FP density	Did not change	Did not change
Basal cell proliferation	Reduced in apical half of FP	Reduced in apical half of FP^3^
Innervation	Maintained, as assessed with NF and P2X3 immunoreactions	Maintained, as assessed with NF and P2X3 immunoreactions^2,3^
CT nerve response to chemicals	Eliminated for all stimuli	Eliminated for all stimuli^2,3^
CT nerve response to tactile stroking	Maintained	Maintained^2,3^
CT nerve response to cold water (4 °C)	Eliminated after 28d sonidegib treatment	Eliminated after 28d sonidegib treatment^2^
**Soft Palate**		
TB morphology	Fewer cells, altered pore structure	Not done
TB count	Reduced to 50% of Vehicle	Not done
**Circumvallate papilla (CV) and Glossopharyngeal (GL) Nerve Responses**		
CV morphology	Depth and wall length maintained	Depth minimally reduced after 48d, wall length maintained
TB pores	Eliminated	Reduced to 20–50% of Vehicle
Basal cell proliferation	Maintained	Maintained
Innervation	Maintained, as assessed with NF and P2X3 immunoreactions	Maintained, as assessed with NF and P2X3 immunoreactions
GL nerve response to chemicals	Eliminated for all stimuli	Substantially reduced for all stimuli; some moderate responses maintained
GL nerve response to tactile stroking	Maintained	Maintained
GL nerve response to cold water (4°C)	Maintained	Maintained

HPI effects are summarized and compared between rat and mouse for fungiform papillae (FP), taste buds (TB), cell proliferation, innervation, and, chorda tympani (CT) nerve responses; soft palate taste buds; circumvallate papilla (CV), taste buds (TB), cell proliferation, innervation, and, glossopharyngeal (GL) nerve responses. All results are from the current paper, except where data are referenced with literature citations.

In anterior tongue FP/TB, posterior tongue CV/TB, and soft palate TB, the effects of HPI with sonidegib are profound in rat, essentially eliminating TB cells and taste responses from CT and GL nerves. Therefore HH regulates taste organs across lingual and oral tissue types. Mechanisms include HPI affects on proliferation, not by a general defect but by decreased proliferation particularly within the apical FP. Decreased proliferation within FP basal epithelial cells after HPI has been reported and TB cell death *per se* has been ruled out as a driving factor in TB cell loss^3,21^. A differentiation defect was noted, also, in the FP apical region^3^.

In contrast to FP, cell proliferation apparently was not altered in the rat or mouse CV. Possibly the overall CV size, extent, and numbers of basal epithelial cells mitigate against decreased proliferation; or perhaps secreted factors from von Ebner glands can maintain the general papilla epithelium but not TB specifically. Further the FP and CV are in very different local tissue contexts and might be differentially sensitive to HH regulatory effects, as in development of taste organs^25^.

Modality specific effects are a key element in our functional data on taste homeostasis. Whereas TB are eliminated in rat anterior (FP) and posterior (CV) tongue, innervation remains within the papillae after HPI with sonidegib. However, even when challenged with stimulation by a range of chemicals, salt concentrations, and high concentration chloride salts, there were no taste responses from the CT or GL. Thus, after inhibition of HH signaling, two major taste nerves, innervating vastly different lingual taste fields, did not respond to lingual chemosensory stimulation in rat.

Notably, however, there were robust responses from the CT and GL when the respective tongue region was stroked with a wooden rod or stimulated with cold water. Therefore, *epithelial* HH signaling, essential in chemosensation, does not apparently have a major role in sustaining lingual somatosensation. After HPI the remaining nerve fibers from the CT or GL are in close apposition to the papilla epithelium, at the basal lamina near former TB locations; and some fibers extend into the papilla epithelium. We propose that these are somatosensory fiber endings or fibers associated with somatosensory organs^2,3^. Although nerve ending types have been described associated with fungiform and filiform papillae, there is no definitive identification or proposal for specific tactile or temperature responsive end organs or fibers from the CT nerve^26^.

Our data demonstrate somatosensation conveyed in CT and GL nerves that process taste sensation, for tactile stroking and temperature modalities but not mediated via TB cells, which have been eliminated after HPI. Notably we recorded responses to stroking the tongue. The responses are sustained during the stroke only, do not adapt but remain regular in discharge, and do not respond to point pressure on the tongue. Stroking is a particular type of somatosensory stimulation, presumably mediated by low threshold mechanoreceptors (LTMRs), innervated in glabrous skin by Aβ fibers and referred to in hairy skin as Aβ field LTMRs^27,28^. In the geniculate ganglion (GG), that contains the soma of CT fibers, there are distinctive lingual receptive fields for neurons that respond to stroking the anterior tongue and/or to cold water stimulation but do not respond to a sustained point pressure directly on the tongue^29^. Therefore the presence of unique subsets of GG neurons committed to taste, touch and/or temperature modalities, and their FP receptive fields, were demonstrated, a proposition reinforced by the current study. Furthermore, a subset of GG neurons committed to taste and somatosensation has been proposed from transcriptome analysis^30^. Most importantly, a unique role for the GDNF-ret pathway in specifying lingual tactile receptors has been demonstrated^31^. The role for GDNF-ret signaling in lingual mechanosensation is the only demonstrated, direct molecular link to touch responses in the CT.

Interestingly, in rat after 28d sonidegib treatment there were no longer responses to a cold water stimulus in the CT, compared to 16d. This is comparable to mouse CT data^2^. However, in our rat GL recordings even after 36d sonidegib treatment there still were responses to cold water, as there were with tactile stimulation. Previous reports have described robust responses to touch and cold water in rat GL nerve responses to CV regional stimulation^32^, but only taste responses had been systematically addressed. It is important to pursue related studies to identify the mechanoreceptors and thermoreceptors in the lingual taste organ periphery, innervated by CT fiber/GG neurons and GL fibers/petrosal ganglion neurons, and the particular neuron subsets that mediate these somatosensory responses in CT and GL nerves, but not within TB *per se*, and that apparently are spared in HH signaling suppression in the epithelium. The panoply of dorsal root ganglion somatosensory types include low threshold mechanoreceptors involved in touch, pressure and vibration, and smaller unmyelinated neurons for nociception, thermoreception, itch and gentle touch^27,33^. These several receptors and mediating neurons are not yet discovered/identified in any detail in lingual receptive fields.

Our data, indicating that somatosensation remains in anterior and posterior tongue after HPI with sonidegib, whereas taste responses are lost, are important to patients treated with HPI drugs who report taste disturbances such as “food tasting bland” and there are “metallic tastes”^34^. Although these taste disturbances have not been systematically defined, clinicians can inform patients that diets emphasizing tactile stimulation and cold might enhance positive reactions to meals.

The innervation that remains in FP and CV after HPI, although robust, cannot maintain TB. However when HPI treatment is discontinued, the TB return/regenerate in FP and CV in mouse^3^. We and others have shown that GG neurons and nerve fibers are a source of SHH in taste organs^3,21,35^. We also reported that after HPI, SHH+ expression remained in the GG soma and furthermore, after HPI there was no decrease in numbers of GG neurons that express SHH^3^. However the GG/CT source of ligand, that remains after HH Pathway Inhibition with sonidegib has eliminated TB and therefore the TB source of SHH, is alone not sufficient to maintain TB in the context of HPI^3^. Therefore a papilla epithelium with integral HH signaling is essential to interact with innervation in maintaining TB, and taste sensation, demonstrated in both anterior and posterior tongue.

Whereas HPI effects on TB homeostasis are severe, there is partial recovery in mouse FP/TB after 16d sonidegib treatment and subsequent periods of no drug for up to 9mo^3^. About 50% of FP acquired a typical TB after recovery; HH signaling was intact within the FP; and, CT responses returned to typical levels. However, when we extended the treatment period to 48d in the current study in mouse, even after recovery periods up to 5–7mo there were no typical TB in FP (summarized in Table 2). We suggest that some TB remnants must remain, as seen in Atypical FP/TB, to enable recovery after HPI. Possibly the SHH expressed in TB remnants can sustain HH signaling in the epithelium, necessary for recovery of TB. In the mouse CV analysis there is TB recovery back to vehicle numbers after sonidegib treatment for 16d^3^ or 48d (Table 2), after 3mo without drug. In these CV tissues there had been retention of TB with or without pores after 16d and 48d sonidegib treatment.

**Table 2:** Taste system recovery from HH Pathway Inhibition (HPI) by sonidegib drug removal.

Taste Effect	Mouse Anterior Tongue	Mouse Posterior tongue
**Recovery from 16 days of HPI with sonidegib**		
Papilla morphology	~50% of FP recovered normal structure^3^	CV depth and wall length maintained^3^
TB count	Recovered in ~50% of total FP^3^	Complete recovery after 40% loss of TB^3^
Nerve response to chemicals	CT nerve responses returned to Vehicle levels^3^	Not done
**Recovery from 48 days of HPI with sonidegib**		
Papilla morphology	No recovery after >95% loss of typical FP structure	CV wall length retained, alterations in depth did not recover
TB count	No recovery after >95% loss of TB	Complete recovery

Recovery effects are summarized and compared between anterior and posterior tongue papillae and TB in mice after 16d or 48d of sonidegib treatment, followed by several months with no drug. All results are from the current paper, except where data are referenced with literature citations.

Here we have addressed HPI effects in rat and mouse, in FP and CV structure and function. From developmental origins^25,36,37^ through adult tissue expressions with particular innervations^11^, there are extensive differences between the anterior and posterior tongue tissues and taste organs. TB environments in FP and CV are in many ways more dissimilar than similar, with TB packed in close contact in troughs of the CV and residing singly in apex of FP. Therefore in our comparison of anterior and posterior tongue fields it is perhaps not surprising that there are differences between species and in the nature of molecular regulation demonstrated with HPI.

We compared our data in the rat *anterior* tongue taste system with data from HPI in mouse^3^ (and see Fig. 8a) and learned that there are species similarities in HH signaling regulation. TB and taste sensation are effectively eliminated in anterior tongue after HPI with sonidegib treatment. Moreover, the apical, conical keratinization in FP and modality-specific effects are similar, indicating that HH signaling effects in FP/TB maintenance and neurophysiological function generalize.

Whereas HPI by drug^2,3^ and *Smo* and *Gli* gene deletions^1,3^ has been studied extensively in mouse FP/CT, the CV/GL had much less attention and in fact there were no recordings from the GL in mouse after HPI. We had reported that effects of the HPI drug sonidegib on TB in the mouse CV were less extreme than in anterior tongue TB^3^. Indeed loss of TB after HPI was reduced to about 25% of Vehicle-treated mice in CV compared to 100% loss in FP. In contrast, this difference in degree of effect was not apparent in rat: in FP and CV essentially all TB were eliminated after 16d sonidegib treatment. To test whether this species difference was apparent in function of the GL nerve that innervates CV and TB, we made electrophysiological recordings in rat and mouse. Echoing the taste organ effects, whereas taste responses were essentially eliminated in the rat GL, they were sustained in the mouse GL at a level of about 6% or greater. Overall, the mouse GL responses to chemicals, a NH_4_Cl concentration series, and a set of chloride salts were still robust after 48d sonidegib. In both rat and mouse there are significant differences in proportions of TB cells that express specific markers and signaling molecules and in the density of cells per TB, which is higher in mouse^38^. There may be indirect effects of these parameters in taste responses after HPI.

A very different TB setting is in the soft palate, an understudied, stratified squamous mucosa with numerous glands where TB do not reside in specialized papillae and where HPI effects had not been addressed. The large proportion of oral TB is most dense at the border of hard and soft palates, the Geschmacksstreifen, and down the midline of the soft palate to the nasopharynx^12^. We found a profound loss, almost elimination, of Typical TB after sonidegib treatment. The remaining TB remnants are distinctive as small clusters of cells at the base of the epithelium with multiple, overlying cell layers. Importantly, a role for HH signaling in maintaining TB of the soft palate demonstrates the breadth of HH regulation across oral TB fields in taste homeostasis.

Our results from rat are important in extrapolation to human patient-reported taste disturbances^19,20,34^. Mouse data would predict residual taste responses based on CV/TB and nerve responses. On the other hand, rat data would predict no lingual taste responses from front *or* back of tongue, or indeed from the soft palate. Data from mice treated with the HPI drug vismodegib, indicated modest loss of CV/TB cells and no loss of taste preferences^23^. It is important, then, to carefully consider use of drug, assessed tissue location, and species when extrapolating HPI effects to the human taste system. Furthermore our data demonstrate that with prolonged sonidegib treatment there is reduced FP taste organ recovery after stopping drug treatment. This suggests that although taste organs should regenerate after HPI, with prolonged suppression not all TB will reconstitute.

In summary, in rat, with well-studied physiology^39^ and extensive use in behavioral taste assessments^40,41^, we have validated not only the HH signaling regulation of anterior taste organs and taste sensation seen in mouse but also extended understanding of this regulation to TB in the soft palate and to posterior tongue function in the GL nerve. We affirmed that HH regulation of taste organs is modality - specific; that is, HPI eliminates TB and neural taste responses from the CT and GL while leaving functional responses to touch and temperature intact. Because effects of HPI in the rat taste system are more profound than those in mouse, and there are differences in murine FP and CV effects, applying HPI rodent data to human taste disruptions should be done with care in considering species.

## Materials and Methods

### Animals, treatments

Adult female Sprague-Dawley rats (Charles River; 190–250 g) or adult male C57BL/6 mice (Charles River; 25–30 g) were used. We did not predict gender effects based on previous studies with male and female mice^1,3^. Procedures followed National Institutes of Health guidelines and were approved by the University of Michigan Institutional Animal Care and Use Committee. We used three or more animals per group; numbers are in figures. The same animals were used for electrophysiology, histology and immunostaining. For morphology several FP were assessed; numbers are in figure legends.

Animals had daily oral gavage for 16–48d (days) with sonidegib (ChemieTek NVP-LDE225) dissolved in vehicle (PEG 400, 75%:5% dextrose in water, 25%) at 20 mg/kg, or vehicle alone^2^. The gavage probe, polyethylene tubing (PE-100: Becton Dickinson) for rats, or 24 gauge, 25 mm (Instech) for mice, by-passed oral tissues for delivery into the stomach. Body weight gain averaged 37% in Vehicle - and 35% in Sonidegib - treated rats over 24 days.

### Surgeries for neurophysiology

*CT nerve in rat*. The rat was anesthetized with an intraperitoneal (I.P.) injection of pentobarbital sodium (50 mg/kg body weight), with supplemental doses as required, secured in a non-traumatic head holder, and placed on a heating pad. The trachea was cannulated and both hypoglossal nerves cut to prevent reflex tongue movements. The CT nerve was dissected by a lateral approach, cut, desheathed and placed on a recording electrode, with a reference electrode nearby. Silicone elastomer (World Precision Instruments) was placed in the dissected nerve cavity to maximize recording stability. The tongue was extended and secured by sutures through the ventral tip.

*GL nerve in rat*. The rat was anesthetized with an I.P. injection of pentobarbital sodium (50 mg/kg body weight), and prepared for dissection as for CT. For access to the CV, an additional incision was made^42^, and the masseter muscle and right half of the mandible removed for oral exposure. Facial vein branches were ligated and tissues separated for access to posterior tongue. The CV was exposed by tension on the lower incisors; sutures through the ventral tongue opened the CV trenches for stimulation. The GL nerve was dissected by a ventral approach^32,42^. Submaxillary and major sublingual glands, posterior belly of the digastric muscle, and tip of the horn of the hyoid bone were removed. The external carotid artery was ligated and cut. The GL nerve was dissected, and the small branch of the pharyngeal GL was cut. Then, the GL nerve was cut, desheathed and placed on a recording electrode, with a reference electrode nearby. Silicone elastomer was applied in the nerve cavity for recording stability.

*GL nerve in mouse*. Mice were anesthetized with Ketamine-xylazine mixture (80–100 mg/kg ketamine; 5–10 mg/kg xylazine, I.P.), maintained with supplemental ketamine doses (80–100 mg/kg). The GL nerve was cut, dissected, desheathed and, placed on a recording electrode with a reference electrode in nearby tissue. The CV was exposed by tension on the lower incisors and sutures through the ventral tongue. A skin incision at the angle of the mouth gave access to the CV.

### Stimuli and stimulation protocols

Reagent grade chemicals, dissolved in distilled water, were: 0.10, 0.50M NaCl, 0.10, 0.50M NH_4_Cl, 0.01N HCl, 1.00M sucrose, 0.04M quinine HCl (QuHCl), 0.10M citric acid, and 0.50M L-Glutamic acid monosodium salt hydrate (MSG); a chloride salt series at 0.50M: NaCl, KCl, NH_4_Cl, CaCl_2_, MgCl_2_; a salt concentration series: 0.05, 0.10, 0.25, 0.50, 1.00M NaCl or NH_4_Cl. Stimuli were applied for 20 s via syringe to the anterior tongue at room temperature or to the CV at 30–36 °C, followed by water rinse at the same temperature for at least 30 s. NaCl or NH_4_Cl was applied three to four times throughout the series to monitor recording stability. The entire stimulus series was repeated. Tactile stimuli were stroking the anterior tongue or CV five times with a wooden rod. The cold stimulus was 4 °C distilled water applied to anterior tongue or CV, with room temperature rinses. Although water at 4 °C could be considered in a ‘noxious’ range for skin sensation^43,44^, we do not equate cold water, at refrigerator temperature, as noxious for oral sensation.

### Neurophysiology

Neural activity was amplified with a Grass P511 preamplifier, displayed on an oscilloscope and monitored by an audio amplifier. Amplified activity was digitized, passed through an integrator circuit, then stored using Spike2 Version 4 Program (Cambridge Electronic Design). Data were quantified by measuring the height of recorded responses above baseline at 5–10 sec after stimulus application^2^.

### Tissue preparation

Animals were euthanized, tongues on mandible were dissected and fixed for 5–7 hrs in 4% PFA^1,3^. Tongues were removed from the mandible, cut anterior to the intermolar eminence, and bisected. One half of the tongue and/or the dissected CV were cryoprotected with 30% sucrose and embedded in O.C.T. for immunostaining. The other half tongue and/or CV were further fixed in 4% PFA for paraffin embedding and H&E staining. The soft palate, including a hard palate segment, was harvested from rats only and fixed overnight at 4% PFA for histology. Tissues were serially sectioned at 6 µm (mice) or 8 or 10 µm (rat) for histology and 10 µm for immunostaining: anterior tongue in sagittal plane; CV in horizontal plane; soft palate in coronal plane.

### Histology

#### Fungiform papillae (FP) and taste bud (TB) quantification

For each half tongue, 600 μm (mice) or 800 μm (rat) of the mid-region of tissue was analyzed, excluding sections on the median furrow or lateral edges^1,3^. In serial sections, FP and resident TB were categorized and counted as (Fig. 1a): (1). Typical FP/TB: The rectangular papilla has a broad connective tissue core covered by a thin epithelium with a single apical taste bud and pore. (2) Atypical FP/TB: The papilla is misshapen with multiple, cornified apical layers that form a pointed cap. TB have reduced cells and lack a taste pore that traverses the cornified layers. (3) Atypical FP/No TB. The Atypical papilla is as described in Category 2 without any discernible taste cell collections.

#### Circumvallate papillae (CV) and taste bud (TB) quantification

Sections were analyzed to measure CV depth and wall length, TB pores and/or TB profiles^1,3^. Depth is calculated as number of sections that include the CV walls times section thickness (6 or 8 µm). Wall length is determined in the middle CV section by measuring the length of each trench and calculating the average. In the middle 10 sections, all TB or remnants of taste cell collections are counted and summed to represent TB profiles; each TB with a complete taste pore is also counted to determine TB pore numbers.

#### Soft palate taste bud quantification

TB were quantified in 1.5 mm of the soft palate from the transition to hard palate, at the ‘Geschmacksstreifen’^12^. The entire width of soft palate, about 6 mm, was included. TB were categorized into (Fig. 3): Typical TB, cells of the TB span the depth of the epithelium and there is a taste pore; Atypical TB, the TB loses its cells, is covered with cornified thick layers, and has no typical pore. Data are represented as total TB and the percentage of Typical and Atypical TB.

### Immunostaining

Immunoreactions were according to routine procedures^1–3^. Antibodies included: SHH ligand: goat anti-SHH (AF464, 0.1 µg/ml, R&D Systems for mice), mouse anti-SHH (sc-365112, 1:500, Santa Cruz Biotechnology for rat); taste cell markers K8, K18, K19^45–48^, with different cytokeratins for optimal immunoreactions in double labeling, K8 (rat TROMA-1, 1:1000, Developmental Studies Hybridoma Bank) for mice, K18 (mouse anti-cytokeratin 18, sc-51582, 1:100, Santa Cruz Biotechnology) or K19 (rabbit anti-keratin 19, ab52625, 1:5000, Abcam) for rat; cell proliferation, Ki67 (rabbit anti-Ki67, NB110-89717SS, 1:5000, Novus Biologicals); innervation, NF (chicken anti-neurofilament heavy, ab4680, 1:5000, Abcam), P2X3 (rabbit anti-P2X3, APR-016, 1:1000, Alomone Labs). SHH and Ki67 reactions included antigen retrieval. Secondary antibodies were Alexa Fluor conjugates 461, 488 or 568.

### Cell Proliferation

For FP, 8–10 papillae per tongue were imaged to count Ki67+, proliferating cells, in Apical (upper half) and Basal (lower half) regions of the FP walls. The basal epithelium of the lateral FP was measured on each side from apex to base and then each side was divided into halves^1,3^ (Fig. 2a, K19/Ki67). Counts were summed to represent Ki67+ cells/FP region. For the CV we qualitatively assessed the three middle papilla sections.

### Statistics

Two-way ANOVA compared differences for each FP type in rat, with main effects of treatment or duration of treatment, and group-wise comparisons with the Tukey’s Honest Significant Difference (HSD). For electrophysiology, CV measures and cell proliferation, and mouse FP recovery where we pooled Vehicles, we used one way ANOVA, with Tukey’s HSD posthoc test. The independent samples t test, with Levene’s test for equality of variance, compared the soft palate taste bud count between treatment durations. Statistical analyses were performed using SPSS Statistics 24 (IBM, USA) software. Data in figures are presented as Mean ± S.E.M. Significance was set at p ≤ 0.05. Exact p-values are in figure legends and results.

## Data Availability

The datasets generated or analysed are available from the corresponding author on reasonable request.
